# Counting the dead to determine the source and transmission of the marine herpesvirus OsHV-1 in *Crassostrea gigas*

**DOI:** 10.1186/s13567-018-0529-7

**Published:** 2018-04-10

**Authors:** Richard J. Whittington, Ika Paul-Pont, Olivia Evans, Paul Hick, Navneet K. Dhand

**Affiliations:** 1 School of Veterinary Science, University of Sydney, Camden, NSW 2570 Australia; 2 Present Address: Laboratoire des Sciences de l’Environnement Marin (LEMAR), UMR 6539 CNRS/UBO/IRD/IFREMER, Institut Universitaire Européen de la Mer, Technopôle Brest-Iroise, 29280 Plouzané, France; 3 Present Address: Department of Agriculture and Water Resources, Canberra, ACT 2601 Australia

## Abstract

Marine herpesviruses are responsible for epizootics in economically, ecologically and culturally significant taxa. The recent emergence of microvariants of *Ostreid herpesvirus 1* (OsHV-1) in Pacific oysters *Crassostrea gigas* has resulted in socioeconomic losses in Europe, New Zealand and Australia however, there is no information on their origin or mode of transmission. These factors need to be understood because they influence the way the disease may be prevented and controlled. Mortality data obtained from experimental populations of *C. gigas* during natural epizootics of OsHV-1 disease in Australia were analysed qualitatively. In addition we compared actual mortality data with those from a Reed–Frost model of direct transmission and analysed incubation periods using Sartwell’s method to test for the type of epizootic, point source or propagating. We concluded that outbreaks were initiated from an unknown environmental source which is unlikely to be farmed oysters in the same estuary. While direct oyster-to-oyster transmission may occur in larger oysters if they are in close proximity (< 40 cm), it did not explain the observed epizootics, point source exposure and indirect transmission being more common and important. A conceptual model is proposed for OsHV-1 index case source and transmission, leading to endemicity with recurrent seasonal outbreaks. The findings suggest that prevention and control of OsHV-1 in *C. gigas* will require multiple interventions. OsHV-1 in *C. gigas*, which is a sedentary animal once beyond the larval stage, is an informative model when considering marine host-herpesvirus relationships.

## Introduction

Viruses are important effectors in seawater as they modulate the vast populations of microscopic plants and animals in the plankton [1] but there are marine viruses that we recognise to be pathogens because they are responsible for epizootics in economically, ecologically or culturally significant taxa. Many of these are herpesviruses, which have been well studied in relation to the prominent diseases they cause in humans, terrestrial animals and birds. Common features include the establishment of life long latent infections which can reactivate, and direct transmission in secretions or lesion exudates through close contact between infected and susceptible hosts [2]. Consequently, in addition to vaccination, the separation of hosts, physical barriers to reduce contact rates and disinfection procedures are recommended to break the direct transmission cycles of herpesviruses and to control the diseases they cause. To what extent can this information be extrapolated to herpesviruses in marine ecosystems?

The recent emergence of microvariants of *Ostreid herpesvirus 1* (OsHV-1) including OsHV-1 µVar in epizootics in Pacific oysters *Crassostrea gigas* has resulted in considerable socioeconomic losses in Europe, New Zealand and Australia [3]. This has stimulated research leading to evidence that OsHV-1 may have different properties compared to some well studied herpesviruses. For example OsHV-1 remains viable for at least 1 week in dried tissues from infected oysters and for about 2 days in artificial seawater [4], periods sufficient for indirect transmission. Furthermore, the virus may be attached to particles, spread indirectly between hosts in plankton and be filtered/ingested [5–8] rather than transmitting directly between hosts. A closely related marine herpesvirus, acute viral necrosis virus (AVNV), was shown to associate with microalgae and to be infectious to scallops through feeding [9].

The source of OsHV-1 in European epizootics probably includes the oyster industry, specifically the unregulated movements of subclinically infected *C. gigas* spat, which are the small oysters obtained by farmers to grow to market size. Peeler et al. [10] reported the results of a questionnaire survey of Irish oyster farmers following a mortality event in 2009: mortality began in recently introduced batches and occurred later in oysters that were already established and the authors believed this observation was consistent with the introduction of a pathogen that then spread. Within the French industry, OsHV-1 µVar infection was widespread among batches of spat sourced from French farmers [11] as well as in translocated wild caught spat [12]. Similarly in New Zealand, OsHV-1 was spread due to industrial oyster movement patterns and lack of biosecurity practices [13]. In 2010 the European Food Safety Authority recommended that measures were urgently needed to minimize the risk of transfer of pathogens with batches of spat [14].

**Figure 1:**
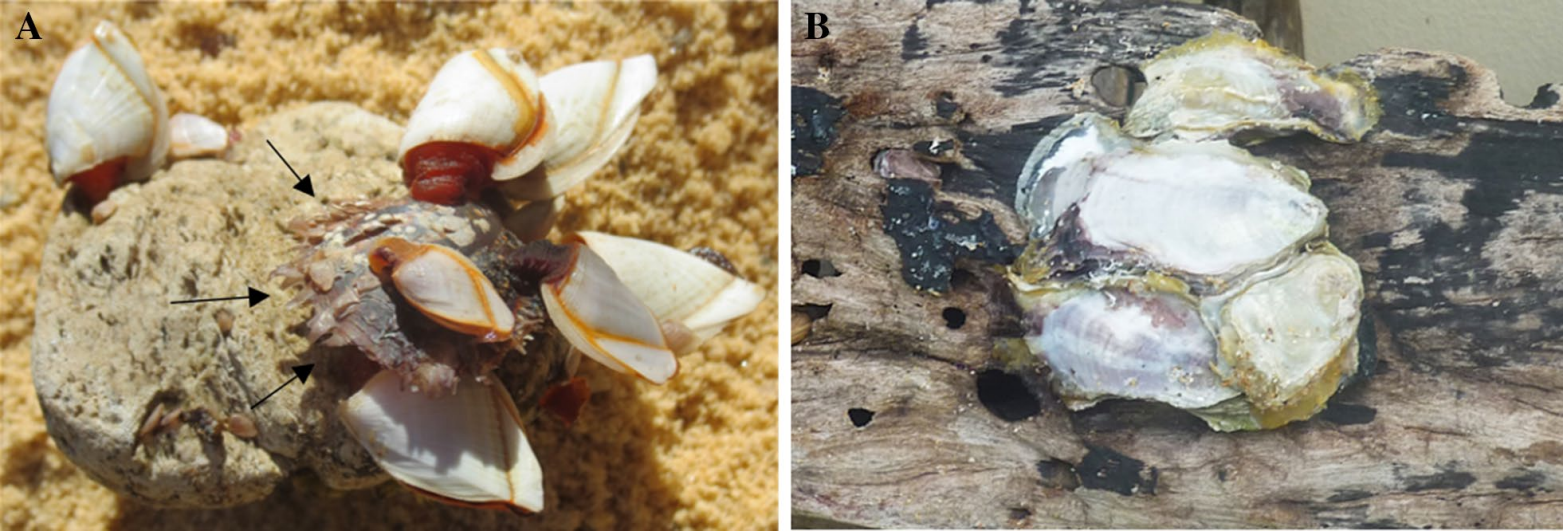
**Examples of rafting of oysters. A** Pacific oyster spat (arrows) and stalk barnacles attached to pumice, January 2014. The pumice is believed to have originated in volcanic activity in the western Pacific ocean. **B** Oysters, species not identified on driftwood, in January 2012. Both items were found newly deposited in summer on an oceanic beach near Wollongong, NSW Australia.

There is no information on how or from where OsHV-1 emerged in New Zealand, and like Australia, mass mortality events caused by OsHV-1 microvariants were unknown prior to 2010. Epidemiological observations in Australia suggested that the sources of the virus for the index cases in the affected estuaries in New South Wales in 2010/2013, Tasmania in 2016 [15–17] and South Australia in 2018 were not the oyster industry. The outbreaks in all three regions commenced adjacent to capital cities (Sydney, Hobart and Adelaide, respectively) and in each case wild oyster populations in bays connected with major commercial shipping ports were clinically affected (Sydney Harbour and Port Botany/Kurnell, Port of Hobart/Derwent River and Port of Adelaide, respectively). In the Wadden Sea in northern Europe, the occurence of OsHV-1 µVar infection in wild *C. gigas* in the absence of a nearby commercial oyster farming industry and where the only introduced oysters were a few 100 kms away suggests that virus transmission in Europe also might occur by means other than commercial oyster translocations; larval *C. gigas* brought in by currents, or oyster biofouling on shipping were suggested as possible sources [18]. Interestingly in 2016 low quantities of OsHV-1 DNA were detected by PCR in *C. gigas* that were attached to the hull of a barge imported into South Australia, a region in which the large *C. gigas* industry is free of OsHV-1 [19]. Shipping ballast water is another mechanism for international dispersal of marine invertebrates and microbes [20]. Oyster larvae may attach to floating objects including driftwood in estuaries and pumice (Figure 1) and have the potential to be transported long distances by oceanic currents [21]. Finally, the international shipment of uncooked seafood and its inappropriate use as bait and burley has been recognised as another means of introduction of exotic aquatic pathogens [22].

Once introduced into an estuary, it has been proposed that there is direct transmission of OsHV-1 to susceptible oysters when the virus is shed into the environment from nearby infected oysters [23]. Mortality in sentinel *C. gigas* in the Thau Lagoon, France was higher in the farming area than outside the farming area, consistent with spread from the farmed oysters with local currents [23–25]. Furthermore, the highest mortality occurred in baskets in which there was very close contact between oysters, and the lowest on ropes to which oysters were cemented with some spatial separation; it was inferred that there was direct transmission between oysters in baskets together with flushing of the infection away from the oysters on ropes [25]. These observations are supported by those from experimental infection models in aquaria in which it is possible to directly transmit the virus from infected donor to naive recipient oysters by cohabitation [8, 12, 26–28] and there is a dose–response effect of the number of donors on infection and disease severity in recipients [8, 29].

However, direct transmission does not seem to fully explain the mass mortality events. In Australia, mortality is usually very unevenly distributed at every scale from the cultivation unit, to the lease, farm and bay [5, 17] and in Europe also the mortality in a given region and time can be highly variable [14, 30]. In the 2013 epizootic in the Hawkesbury River estuary Australia, transfers of infected oysters did not lead to mass mortality in nearby oysters, despite very close contact among intensively cultivated oysters [16].

An understanding of the source of a virus and the means of its transmission is important because it can influence the way a disease is prevented and controlled [6]. For example, certification of hatcheries for pathogen freedom prior to translocation of spat would prevent disease outbreaks if hatcheries were the main source of that pathogen. Reducing the density of susceptible hosts or infected sources can reduce direct animal to animal transmission of pathogens [31], but can be costly to implement and may be impossible in some situations. Partial destocking in the face of an outbreak is often recommended as a generic disease control measure in aquaculture but would be pointless and economically disadvantageous if the infection risk is the off-farm environment and the disease is not directly transmitted. For these reasons evidence on the type of epizootic is important because it can identify the source and whether the virus is transmitted directly or indirectly.

There are two main types of epizootics, point-source and propagating [32, 33]. In a point-source epizootic many susceptible hosts are infected indirectly from a common source external to the population; a point source may be a single event, episodic or continuous, which can result in different patterns of mortality over time. In contrast in a propagating epizootic, hosts are directly infected from others within the population, and the cases occur over successive incubation periods. A population may become infected from a point source, and then the pathogen may propagate between remaining susceptible individuals by direct contact [34]. For pathogens that transmit directly there should be a logical sequence of cases based on their likelihood of contact (for example, based on duration of contact, and/or proximity) and the incubation period.

The aim of this study was to use mortality data obtained from experimental populations of *C. gigas* during natural epizootics of OsHV-1 disease in order to develop better understanding of the source and evolution of an epizootic. We used qualitative analysis, compared actual mortality data with those generated using a Reed–Frost model of direct transmission and analysed incubation periods using Sartwell’s method to test for the type of epizootic. We concluded that outbreaks were initiated from an unknown environmental source and even though direct transmission may occur between larger oysters if they are in close proximity, point source exposure and indirect transmission were more important. Prevention and control of OsHV-1 in *C. gigas* will require multiple interventions.

## Materials and methods

### Definitions

#### Direct and indirect transmission

In this paper the terms direct and indirect transmission are taken from Thrusfield [32] and Webb et al. [33]. Direct transmission does not require any intermediary vehicle or vector, arises from close physical contact with the infected host or its secretions which may pass 1–2 m between infected and susceptible hosts. Indirect transmission means that a vehicle, which could include food, is required, and allows for the possibility of a living vector to move the pathogen sometimes over distances.

#### Group

**Figure 2:**
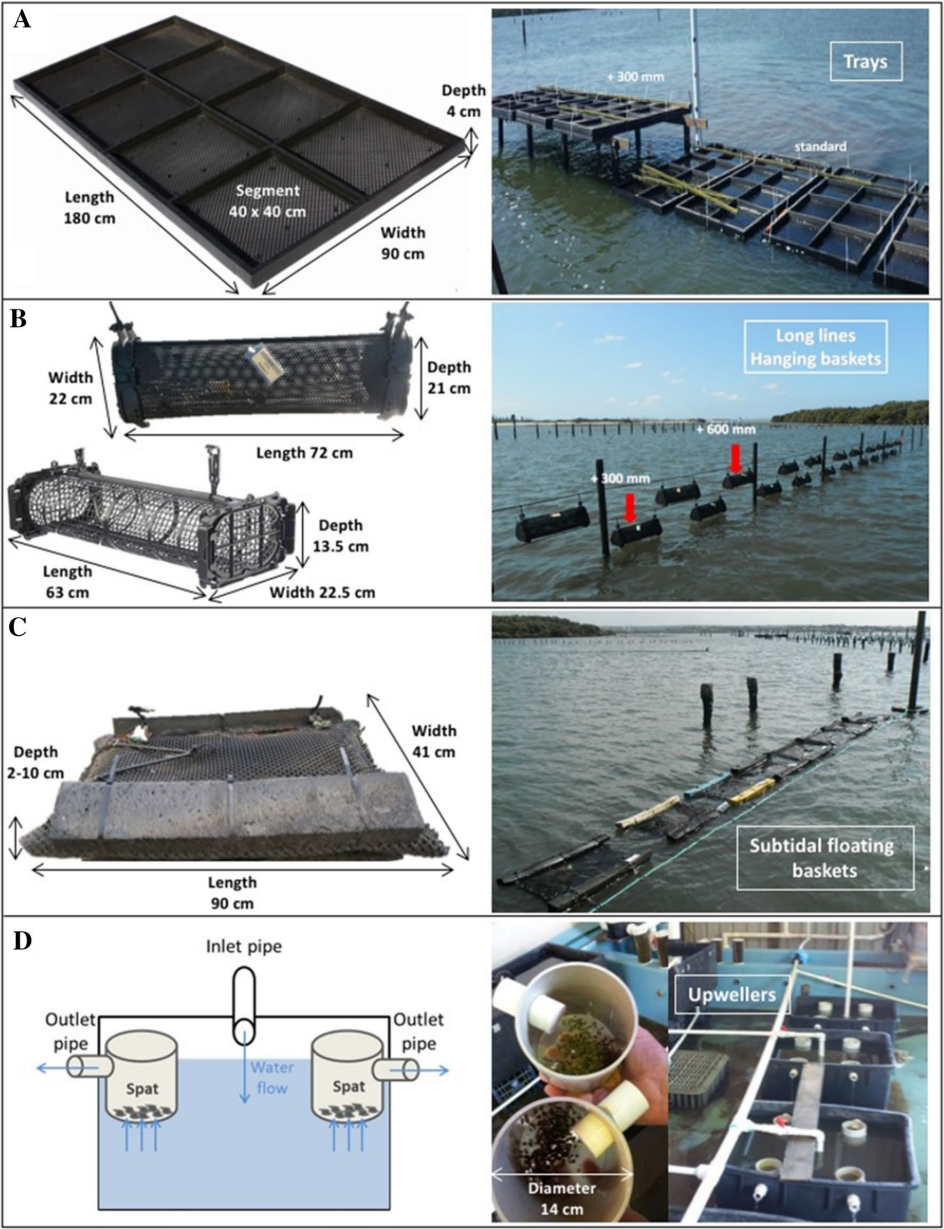
**Dimensions and arrangement of cultivation equipment. A** Intertidal trays which were at two heights, standard and + 300 mm; **B** Intertidal baskets which were at two heights, + 300, + 600 mm; **C** Subtidal (floating) baskets; **D** upwellers.

In the experiments described below a group was comprised of all baskets (*n* = 24 in Experiments 1 and 2; *n* = 12 in Experiment 3) on a long-line or all segments (*n* = 8) of a tray (Figure 2).

#### Case definition

The unit of interest for many of the analyses was the smallest delineated cultivation structure, that is a basket of oysters or a segment of a tray of oysters (see below). A mortality event (i.e. a case) in a unit was defined at a given observation time when ≥ 10% of the oysters that were alive at the previous observation (after removal of samples, if done) had died and where oysters sampled from the group on one or more occasions contained ≥ ~10^4^ viral DNA copies per mg of tissue, which is a level consistent with death due to OsHV-1 infection [35]. Intervals between observations varied between experiments (see below). The unit of interest for analyses of incubation period using Sartwell’s model and Reed–Frost models (see below) was the individual oyster. All dead oysters within baskets, trays and upwellers where OsHV-1 was detected were assumed to have died due to OsHV-1 infection.

### Sources of mortality data

Existing data from previously described field trials conducted at sites A, B, and C in Woolooware Bay in the Georges River estuary, NSW were collated. Cultivation units (tray segments or baskets) from these trials were aggregated into 37 groups for analysis and are described as Experiments 1, 2 and 3 below (Table 1). Data for mortality events in upwellers located at Mooney Mooney on the Hawkesbury River estuary, NSW are described below as Experiment 4. The scale and arrangement of trays, baskets and upwellers is shown in Figure 2 while geographic locations are in prior publications [16, 36]. All of the cultivation equipment had been cleaned and stored dry prior to use for a period longer than that required to inactivate OsHV-1 [4].

**Table 1:** **Mortality events for groups in Experiments 1, 2 and 3 where the unit of interest was the individual tray segment or basket on a long line**

Experiment	Site	Age of oysters (months)	Length of oysters (mm, mean ± sd)	Group	No. of units (baskets or tray segments)	No. of oysters per unit	% units affected	Days of onset	No. units affected on each onset day	Final mortality % within units (range among units)
Baskets										
1	A	8 14	37 ± 4; 73 ± 16 49 ± 3; 79 ± 12	1	24	50	100	33, 40	16, 8	23.8–97.6
1	C	8 14	37 ± 4; 73 ± 16 49 ± 3; 79 ± 12	2	24	50	33	11, 19, 26	2, 5, 1	0–95.2
2	B	8 17	30 ± 9; 48 ± 17 59 ± 9; 69 ± 11	3	24	50	88	11, 19	11, 10	8.5–100
2	C	8 17	30 ± 9; 48 ± 17 59 ± 9; 69 ± 11	4	24	50	88	11, 19, 26	15, 5, 1	4.3–100
3	A	9.5	50 ± 5	5	12	90	75	40, 47, 56, 76, 89, 174	1, 1, 3, 2, 1, 1	0–88.9
3	A	9.5	50 ± 5	6	12	90	100	11, 19, 26, 33, 47, 174	1, 1, 3, 5, 1, 1	31.1–100
3	A	9.5	50 ± 5	7	12	90	100	26	12	32.2–100
3	B	9.5	50 ± 5	8	12	90	83	61, 76, 89, 105, 147	1, 4, 1, 1, 2	0–100
3	B	9.5	50 ± 5	9	12	90	100	47, 56, 61, 76, 118	1, 2, 1, 5, 3	32.2–80.0
3	B	9.5	50 ± 5	10	12	90	92	47, 56	5, 6	10.0^a^–80.0
3	C	9.5	50 ± 5	11	12	90	92	11, 118, 147	2, 1, 8	5.6–90.0
3	C	9.5	50 ± 5	12	12	90	92	47, 118, 147, 174	1, 3, 6, 1	6.7–77.8
3	C	9.5	50 ± 5	13	12	90	92	47, 56, 61, 76, 174	2, 3, 2, 3, 1	0–80
Trays										
3	A	17.5	92 ± 6	14	8	40	88	76, 174	2, 5	7.5–30.0
3	A	17.5	92 ± 6	15	8	40	88	33, 174, 214	1, 3, 3	12.5^a^–30.0
3	A	17.5	92 ± 6	16	8	40	100	26, 174	7, 1	25.0–57.5
3	A	17.5	92 ± 6	17	8	40	100	26	8	25.0–52.5
3	A	9.5	50 ± 5	18	8	40	100	33, 174	3, 5	27.5–60.0
3	A	9.5	50 ± 5	19	8	40	100	11, 19, 26	1, 1, 6	22.5–82.5
3	A	9.5	50 ± 5	20	8	40	100	11, 19, 26	5, 2, 1	45.0–80.0
3	A	9.5	50 ± 5	21	8	40	100	11, 19	2, 6	55.0–100
3	B	17.5	92 ± 6	22	8	40	88	56, 147, 174	3, 1, 3	17.5–35.0
3	B	17.5	92 ± 6	23	8	40	100	47, 56, 174, 214	1, 5, 1, 1	22.5–40.0
3	B	17.5	92 ± 6	24	8	40	100	33, 47, 147	1, 6, 1	30.0–50.0
3	B	17.5	92 ± 6	25	8	40	100	40, 47	2, 6	40.0–55.0
3	B	9.5	50 ± 5	26	8	40	100	47, 56	7, 1	32.5–65.0
3	B	9.5	50 ± 5	27	8	40	100	47, 56, 76	3, 4, 1	32.5–60.0
3	B	9.5	50 ± 5	28	8	40	100	33, 40, 47	5, 1, 2	30.0–70.0
3	B	9.5	50 ± 5	29	8	40	100	33, 40, 47	1, 4, 3	45.0–72.5
3	C	17.5	92 ± 6	30	8	40	88	47, 174, 214	2, 3, 2	7.5–42.5
3	C	17.5	92 ± 6	31	8	40	75	174	6	7.5–37.5
3	C	17.5	92 ± 6	32	8	40	88	47, 76, 147, 174	2, 1, 2, 2	12.5^a^–45.0
3	C	17.5	92 ± 6	33	8	40	100	33, 40, 47, 56, 76, 147	2, 1, 2, 1, 1, 1	30.0–47.5
3	C	9.5	50 ± 5	34	8	40	100	33, 47, 56, 61, 174	2, 1, 2, 1, 2	37.5–75.0
3	C	9.5	50 ± 5	35	8	40	100	33, 47, 76, 174	5, 1, 1, 1	32.5–65.0
3	C	9.5	50 ± 5	36	8	40	100	33, 40	5, 3	55.0–70.0
3	C	9.5	50 ± 5	37	8	40	100	33, 40, 56	6, 1, 1	42.5–65.0

A case was defined as mortality in a basket or tray segment affecting > 10% of oysters between two consecutive observations, and confirmation of the presence of OsHV-1 by quantitative PCR. The final mortality among individual oysters in baskets or tray segments in the group is also shown.

Mortality data from Hick et al. [38] and Whittington et al. [37].

^a^One unit in this group had ≥ 10% cumulative mortality due to occasional mortalities over the duration of the trial, but did not experience ≥ 10% new mortality between any two observations.

#### Experiments 1 and 2

These were conducted in 63 × 22.5 × 13.5 cm (length × breadth × height, L × B × H) plastic baskets (Seapa, Edwardstown, Australia) hanging on intertidal long-lines (Figures 2B) [37, 38]. Experiment 1 commenced in February 2014 while Experiment 2 commenced in January 2015. There were four oyster age–size combinations in each experiment, but each basket contained only one age–size category and basket position on the long-lines was randomised [38].

#### Experiment 3

Oysters were deployed in October 2012 in eight 180 × 90 × 4 cm (LxBxH) intertidal trays (Tooltech, Brisbane, Australia) at each site [37]. Each tray had eight 40 × 40 × 4 cm (L × B × H) internal segments formed by solid dividers (Figure 2A). At each site there were also twelve 72 × 22 × 21 cm (L × B × H) plastic baskets (BST, Cowell, Australia) on each of two intertidal long lines (Figure 2B), and twelve 90 × 41 × 2–10 cm (L × B × H) plastic floating pillow baskets on a subtidal long-line (Figure 2C). The exact location and orientation of each tray and basket was conserved throughout the experiment, except during inspections.

#### Experiment 4

Seven trials commenced between April 2013 and May 2014 using spat that were held in 14 × 10 cm (height × diameter) upwellers (Figure 2D) supplied with estuarine water from the Hawkesbury River, or in small mesh envelope within a control plastic basket (Seapa) floating in the river [6]. There were 500 spat per upweller and 2000 spat in the control basket.

#### Experimental oysters

The age and size of oysters in each group in Experiments 1, 2 and 3 is shown in Table 1. Spat in experiment 4 were 2.5–6.5 months old, approximately 5–10 mm length (range) among the trials. All oysters in Experiments 1, 2, 3 and 4 were single seed triploid stock from a hatchery in Tasmania (Shellfish Culture), a source certified to be free of OsHV-1 by the government authority since 2011. Oysters were tested also at the University of Sydney prior to use in each trial. OsHV-1 was not detected in Tasmania until January 2016, which was after these experiments ended.

### Counts and cause of mortality

Bivalves have a hard shell that opens when the animal dies, exposing the soft tissues which disintegrate and are removed rapidly by scavengers to leave an empty shell. These were counted along with freshly dead individuals to determine mortality rates. The association of mortality with OsHV-1 was confirmed in each experiment by testing oyster tissues collected at each time point using a quantitative polymerase chain reaction (qPCR) [6, 37, 38]. In Experiment 1 baskets were inspected on days 18, 33, 46, 60, 74, 89 and 104. In Experiment 2 inspections were on days 14, 28, 42, 56, 70 and 84. At each time in both experiments dead oysters were counted and removed and, a selection of these were sampled for OsHV-1 testing. In Experiment 3 all oysters were inspected individually and mortality was recorded on days 11, 19, 26, 33, 40, 47, 56, 61, 76, 89, 105, 118, 147, 174, and 214; dead oysters were removed and random samples of live oysters were also collected at each time (except days 56, 89, 105, 118) for OsHV-1 testing. In Experiment 4 mortality counts and samples were obtained from each treatment each day.

### Qualitative assessment of direct and indirect transmission

Five criteria were used to qualitatively assess observed mortality data to infer whether direct transmission from oyster to oyster within a unit or between units or indirect transmission might have occurred:

1. *Time of onset of cases among units in a group* Counts of dead oysters were entered in a spreadsheet (Microsoft Excel) and conditional (IF, and IF (AND)) functions were used to create binary variables to classify the mortality level in each unit at each observation time as < 10% (0, not a case) or ≥ 10% (1, a case). Conditional functions were then applied to identify the observation time when a case was first observed. Synchronous onset of cases was defined to be when all affected units in a group had the same onset day. Asynchronous onset of cases was defined to be when units in a group had different onset days.
2. *Total cumulative mortality within a unit* The number of dead oysters within each unit was counted, a presumption being that effective direct transmission between in-contact individuals of similar susceptibility would result in high mortality within a unit by the end of each experiment.
3. *Logical spatiotemporal patterns of spread between units* To assess whether asynchronous onset might be explained by direct transmission from oysters in one unit to oysters in another unit following an initial infection of only some units in a group, orderly progression of mortality between units was assessed, ignoring potential impacts of hydrodynamics. For baskets this was done by checking for ordinal patterns of cases (onset of mortality) in sequentially numbered and positioned baskets. Evidence for transmission between baskets was defined to be when the interval between days of onset for adjacent units was within one hypothetical incubation period (a conservative value of 10 days was allowed, Table 2). To facilitate this the days of onset for units in each group of baskets were graphed to enable visualisation of ordinal progression. Similarly, in trays there was a rectangular grid of 8 segments; each segment was adjacent to at least three other segments. This could lead to orderly patterns if there was direct transmission from segment to segment; a hypothetical example is shown in Figure 3. Such patterns were assessed by visual examination of plots of epidemic curves for segments within trays.

**Table 2:** **Assumptions for a Reed Frost model of an OsHV-1 outbreak in** ***C. gigas*** **due to introduction of infected oysters**

Parameter	Values used	Comment	Reference
Population size of susceptible oysters (S)	40, 50, 90, 500, 2000 100 000, 1 000 000	For tray segments, baskets and upwellers Relevant to commercial spat cultivation	This paper
Prevalence at the start %	0.1, 1, 10	Infected oysters introduced into the population	
Incubation period days	1, 2, 3, 7	Spat: 1–4 days median Juvenile to adult: 2–3 days for intramuscular injection; up to a few days longer for cohabitation	This paper Schikorski et al. [28] Schikorski et al. [55] Paul-Pont et al. [43] Evans et al. [8]
Probability of effective contact (p)	0.001–0.1	No known values in sedentary aquatic hosts

**Figure 3:**
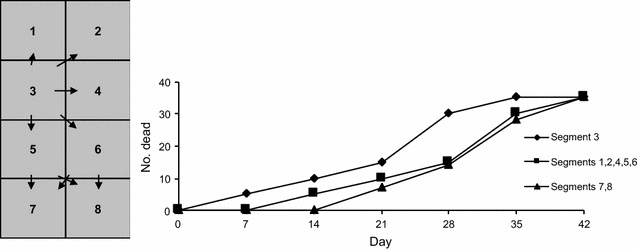
**Hypothetical patterns of direct transmission of OsHV-1 between oysters in different segments of a cultivation tray.** The incubation period is 7 days. Unit 3 is affected initially. The immediately adjacent units are the next ones to show signs of disease. Units 7 and 8 are the last ones to become affected.
4. *Number of units affected over time* The number of new units affected on successive days of onset was determined, assuming that one unit would affect at least one more unit in the group. If effective direct transmission occurred between units, the number of affected units in a subsequent event would be equal to or greater than that in the previous event, until susceptible oysters in units were exhausted.
5. *Final proportion of affected units in a group* The number of affected units in each group was assessed at the end of each experiment, assuming that effective direct transmission would result in many of the units in a group becoming affected due to their close proximity.

### Analysis of incubation periods using Sartwell’s model

Mortality data from Experiment 4 were structured and analysed using Sartwell’s model of the distribution of incubation periods (IPs) to determine the type of epizootic. Only the mortality counts from Experiment 4, which were obtained daily, could be used because of the likely short incubation period for OsHV-1. According to this model, the incubation periods from a point source outbreak follow a log normal distribution [39, 40]. Briefly, the dates of the outbreak and cumulative mortality count over time were extracted for each outbreak. There were 2000 oysters in a single control basket in the river and 500 oysters in each of 4 replicate upwellers in all other groups. The total mortality count across all 4 replicates was used for the latter groups. Assuming the day before the start of the mortality (after detection of OsHV-1 in the group to ensure specificity, as a few oysters died prior to this) as day 0, the dates were converted into days since the start of the outbreak which were then log transformed to calculate log time. Daily mortality percent was calculated by dividing the number of dead oysters on a given day of the outbreak with the cumulative mortality count on the last day of the outbreak. Estimation of the median, standard deviation (SD), dispersion factor (DF) and confidence limits (CL) of the incubation period based on the median IPs and DFs were calculated as suggested by Sartwell by fitting a linear regression model of cumulative mortality percent on log time since the start of the outbreak, which ignored any mortality in the group prior to first detection of OsHV-1, using formulae below.

$${\text{Median IP }} = {\text{ Exp }}\left( {\left( {0. 5{-}{\text{b}}_{0} } \right)/{\text{b}}_{ 1} } \right)$$

$${\text{SD IP }} = \, \left( {\left( {\left( {0. 8 4 1 3 4 4 7 4 6- {\text{b}}_{0} } \right)/{\text{b}}_{ 1} } \right) \, - \, \left( {\left( {0. 1 5 8 6 5 5 2 5 4- {\text{b}}_{0} } \right)/{\text{b}}_{ 1} } \right)} \right)/ 2$$

$${\text{DF }} = {\text{ Exp }}\left( {\text{SD IP}} \right)$$

$$9 5\% {\text{ lower CL IP }} = {\text{Median IP}}/\left({{\text{DF}}^{ 2} } \right)$$

$$9 5\% {\text{ upper CL IP }} = {\text{ Median IP }}*\left( {{\text{DF}}^{ 2} } \right)$$

The parameters b_0_ and b_1_ are the intercept and the slope of the linear regression model of cumulative mortality percent on log time, respectively; and Exp is the exponentiation function.

### Assessment of direct transmission between individual oysters using a Reed–Frost model

A simple Reed–Frost model [32, 41] was built using Excel (Microsoft) to assess direct transmission between individual oysters within individual cultivation units. The model used the formula C_t+1_ = S_t_(1 − q^Ct^) to describe the number of cases (C) at this time point (t + 1), based on the number of susceptible oysters at the previous time (S_t_) and the probability of an infected oyster not making effective contact with a susceptible oyster (q) (where q = 1 − *p* and *p* is the probability of effective contact). The model assumes a closed population, direct transmission, cases being infectious only within one time interval and a fixed probability (*p*) of a case transmitting infection through effective contact [41]. The time interval between each stage of the epidemic was the incubation period, and to fit the model structure oysters in the population were divided into the categories of cases, susceptible, or dead, the last being equivalent to the immune category of the classic model [42]. The total number in each category was calculated after each time interval to produce a graph of cumulative mortality. The parameters needed to create the model were based on published studies (Table 2), except for *p*, which is unknown. A range of values for incubation period, *p,* the number of infected oysters at the start and the population size (S_0_) were used by trial and error in order to try to obtain curves that resembled those observed from the field experiments, assessed graphically.

## Results

### Time of onset of cases among units in a group

**Figure 4:**
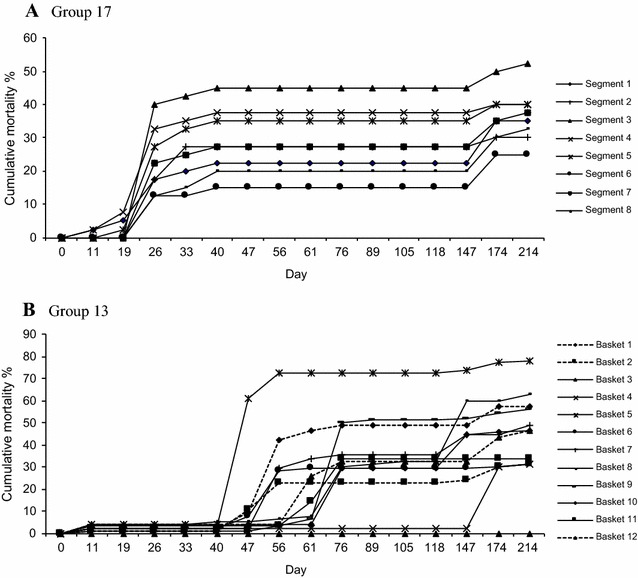
**Cumulative mortality curves for each of the units in a group. Data are % cumulative mortality. A** Eight units being the segments in a cultivation tray (Group 17); synchronous onset of mortality among units; n = 40 oysters per unit. **B** Twelve units being the baskets on a long-line (Group 13); asynchronous onset of mortality among units; *n* = 92 oysters per unit. In both **A** and **B** there is incomplete mortality in each unit and variable final mortality rates between units.

Of the 37 groups, 3 had units with synchronous onset of mortality i.e. 1 day of onset among units, suggesting that they were all exposed at the same time. A representative example of synchronous mortality is shown in Figure 4A. The remaining 34 groups had units with asynchronous onset; there were between 2 and 6 different days of onset (Table 1) and a representative example is presented in Figure 4B. Different days of onset could represent different exposure events from a point source, or direct transmission between units. Intervals between days of onset ranged from 1 week to several months. For example in Group 22 there were 3 separate mortality events but given that the time intervals between events at day 61 and day 118 exceeded the hypothetical incubation period, the mortality could not be explained by direct transmission between segments. This is assessed further below.

### Total cumulative mortality within a unit

The final cumulative mortality of oysters in individual cultivation units within the groups varied substantially (Table 1). For example mortality per basket ranged from 4.3 to 100% among the 24 baskets in Group 4, while mortality per tray segment ranged from 23 to 83% among 8 tray segments in Group 19. Among the 37 groups, only 6 contained units in which all the oysters died, and in just over half the groups (19), no more than 70% of oysters died in any unit. Therefore direct transmission between oysters in a unit was inefficient.

### Logical spatiotemporal patterns of spread between units

**Figure 5:**
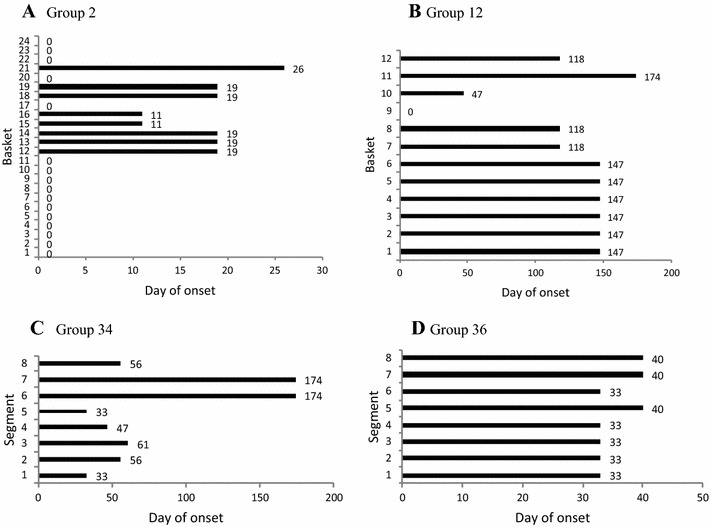
**Day of onset of mortality among units in groups at Site C. A** Group 2, 24 baskets on a hanging long line. There are unaffected baskets between those affected at days 11, 19 and 26, which is evidence against direct transmission between units. Basket 14 affected at day 19 is adjacent to basket 15 affected at day 11, which is consistent with direct transmission because the interval between days 11 and 19 is within one hypothetical incubation period. **B** Group 12, 12 baskets on a hanging long line. There are no examples of direct transmission from basket 10, which was the first one affected, or subsequently from baskets 7, 8 or 12. **C** Group 34, 8 segments of a tray. There are no examples of direct transmission from segment 5, the first one affected. **D** Group 36, 8 segments of a tray. Direct transmission may have occurred between segments affected at day 33 and day 40.

Orderly progression of mortality between baskets was assessed using graphical presentation of the data; examples are shown in Figure 5. In Experiments 1 and 2 the first and last days of onset among units were within one hypothetical incubation period for all 4 groups so that direct transmission from unit to unit could have occurred. However, in Experiment 3 the range of days of onset among units was 11–174 days after deployment of oysters. In 9 of 13 (69%) groups of baskets the onset of mortality between some adjacent units was within a 10 day hypothetical incubation period (Figure 5A) but in 31% of groups there was no such evidence (Figure 5B). For trays, the onset of mortality between some adjacent segments also was within a hypothetical 10 day incubation period in 12 of 24 (50%) groups (Figures 5C and D). Of the 34 groups of baskets and trays with asynchronous onset of mortality among units, in 9 (26.5%) there was some evidence for direct transmission between units, in 12 (35.3%) there was evidence both for and against direct transmission between units (Figure 5A) and in 13 (38.2%) there was no evidence of direct transmission between units (Figure 5B).

### Number of units affected over time

Within each of the 34 groups with asynchronous onset among units, there was a great deal of variation in the number of units affected each time with no clear pattern of escalation (Table 1). For example the number of units affected on each of the three onset days in Group 4 was 15, 5 and 1, and in Group 20 it was 5, 2 and 1. Where the first mortality event affected less than half of the units in a group (15 groups) so that there were still many units with susceptible oysters and therefore an opportunity still existed for direct transmission between units, the number of additional units affected within an incubation period of 10 days was the same as before for 3 groups (Groups 5, 6, 19), lower than before for 3 groups (Groups 3, 33, 34) and higher than before for 9 groups (Groups 2, 9, 10, 13, 21, 23, 25, 27, 29). But in subsequent events that occurred within 10 days, the numbers affected fluctuated up or down and there was no clear pattern (Groups 5, 6, 9, 13, 33, 34). Using this criterion there was equivocal evidence for direct transmission between units.

### Final proportion of affected units in a group

In two of the groups with synchronous onset of mortality (Groups 7, 17), all units were affected and in the third (Group 31), 75% of units were affected by the end of the experiment (Table 1). For 20 of the 34 groups with asynchronous onset of mortality, all units in the group were affected, but in the remaining 14 groups there were still some unaffected units. In Group 2 only 8 of 24 units were affected. Based on this criterion, in 15 of the 37 groups direct transmission did not occur between all of the units.

### Incubation period and type of epizootic from Sartwell’s model

**Figure 6:**
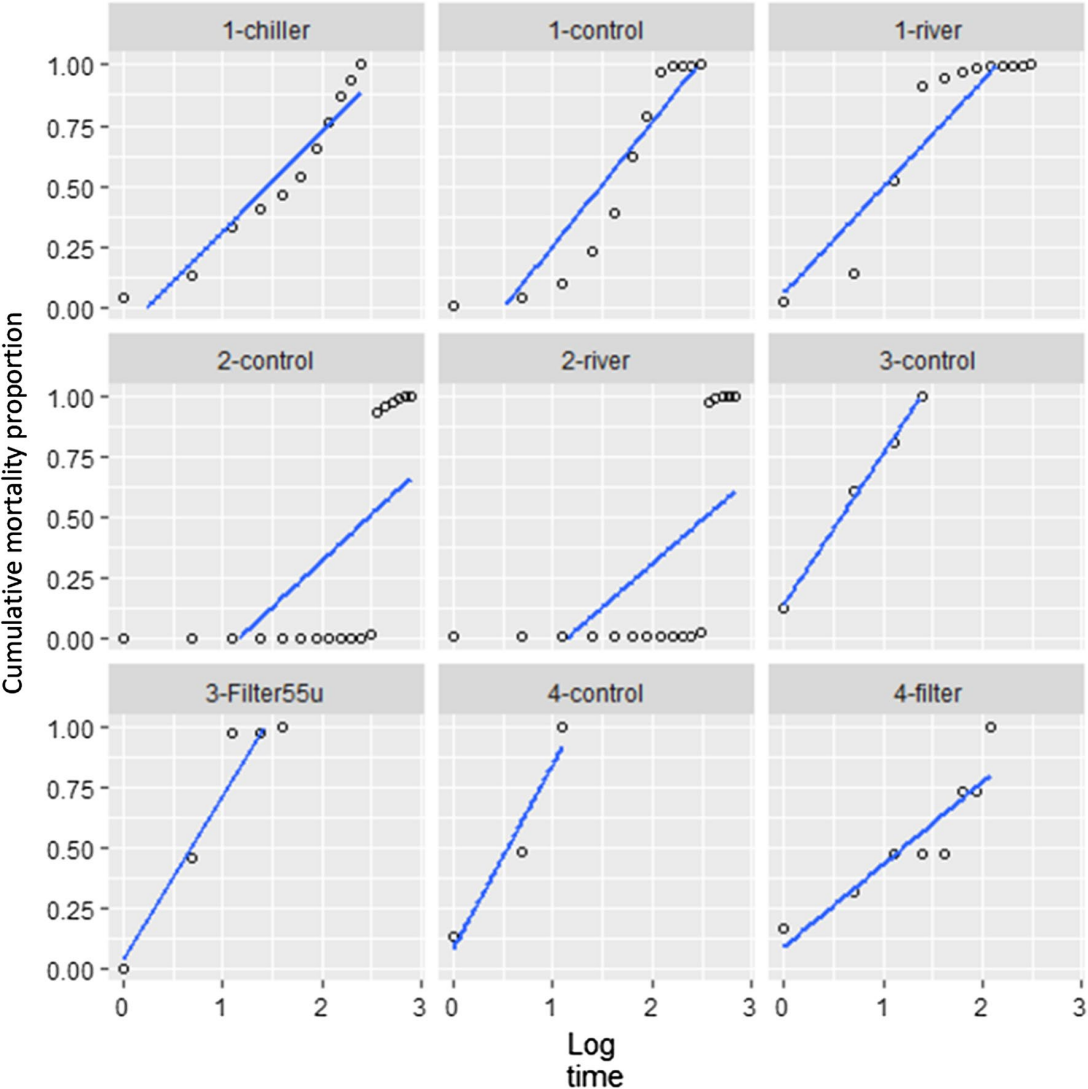
**Mortality of spat in upwellers from nine treatment groups in Experiment 4**. Scatter plots and regression lines between cumulative mortality proportion and log time since the start of the outbreak; according to Sartwell’s model, a linear relationship is consistent with a point source epizootic. Trial numbers and treatments correspond to Table 3.

Daily mortality observations for epizootics in small spat in upwellers and baskets were available from 9 treatment groups with substantial mortality among Trials 1, 2, 3 and 4 in Experiment 4. The median incubation periods estimated based on Sartwell’s model are presented in Table 3 and ranged from 1.1 to 4.4 days among these outbreaks. Scatter plots of cumulative mortality and log time are presented in Figure 6 and suggest linear association consistent with point source outbreaks [39, 40] in all outbreaks except the two groups in Trial 2. The results suggest that the spat in each trial were mostly infected from an external source rather than from one another.

**Table 3:** **Incubation periods for OsHV-1 infection in spat in upwellers, in which the unit of interest was the individual oyster, determined from mortality data from Trials 1, 2, 3 and 4 in Experiment 4**

Trial	Group	Final cumulative mortality %	Intercept	Slope	R-square	Incubation period (days)				
						Median	SD	DF	Lower CL	Upper CL
1	Chiller	42.3	− 0.10	0.41	0.93	4.28	0.83	2.29	0.82	22.39
1	Control	100	− 0.26	0.51	0.87	4.39	0.67	1.95	1.16	16.67
1	River	97.2	0.06	0.44	0.85	2.73	0.78	2.18	0.57	12.97
2	Control	87.8	0.46	0.28	0.49	1.14	1.23	3.42	0.10	13.35
2	River	59.3	0.29	0.49	0.68	1.52	0.70	2.01	0.38	6.13
3	Control	99.9	0.14	0.62	1.00	1.78	0.55	1.73	0.59	5.34
3	Filter55u	60.2	0.04	0.67	0.93	1.98	0.51	1.66	0.71	5.47
4	Control	100	0.09	0.76	0.94	1.72	0.45	1.57	0.70	4.23
4	Filter	100	0.09	0.34	0.83	3.28	1.00	2.72	0.44	24.36

Mortality data from Whittington et al. [6].

### Assessment of direct transmission between individual oysters using a Reed Frost model

**Figure 7:**
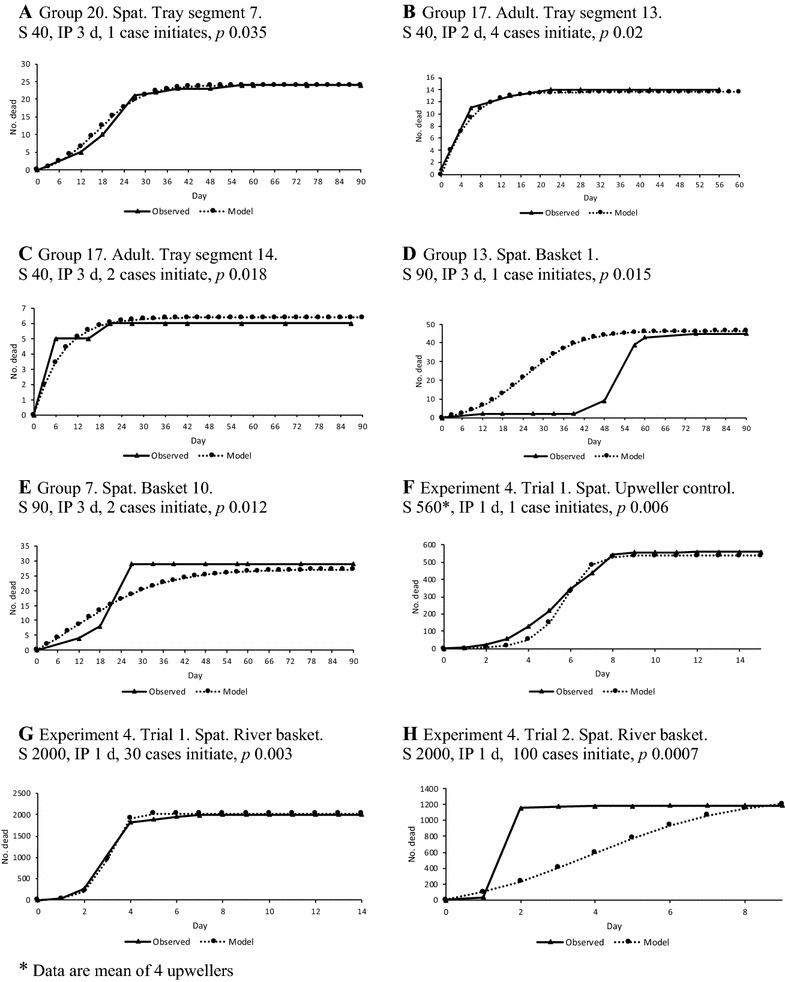
**Observed mortality and that predicted in Reed Frost models.** Representative examples are shown of observed cumulative mortality associated with OsHV-1 infection in different groups of oysters and cumulative mortality predicted in simple Reed-Frost models. **A**, **B**, **C** Individual segments of intertidal trays; **D**, **E** Intertidal baskets; **F** Upweller; **G**, **H** Subtidal (floating) baskets. The size of the population of susceptible oysters (S), the incubation period (IP) in days (d), the number of infected oysters that initiate the outbreak and the probability of effective contact *p* are shown for each model.

Using the actual values for the number of susceptible oysters and reasonable assumptions about the number of cases that initiate an outbreak it was possible to create realistic cumulative mortality curves, i.e. resembling the observed data, for populations of 40 susceptible oysters, the number present in tray segments, regardless of the final mortality being high, moderate or low (Figures 7A–C). In order to have curves with appropriate slope, points of inflection and to have some surviving oysters at the end, the starting number of affected oysters to initiate the epizootic usually needed to be < 5% but was as high as 10% of the population, and values required for *p,* the probability of effective contact were between about 0.01 and 0.04. These findings suggest that direct transmission may have occurred in these populations of oysters, which were spat and adults ~50–90 mm in length. It was not possible to obtain modelled epidemic curves that resembled the observed data for successive mortality events which occurred months apart in a given cultivation unit, such as those illustrated in Figures 4 and 5; to do so would have required creating a separate model for each event, which suggests that the two successive events were initiated independently. Similarly it was also not possible to model the common mortality pattern that was seen in the baskets with 90 spat ~50 mm in length, where there was a delay between the onset of a low level of mortalities that were confirmed to be associated with the virus [37], and the main mortality event (Figures 7D and E). Unless *p* was set to very small values, 0.006 or less, it was not possible to obtain realistic mortality curves for populations of ~500 small spat (~ 5 mm length) in upwellers (Figure 7F) or of ~2000 of these spat confined in baskets (Figure 7G). Such small values of *p* are inconsistent with efficient direct transmission, meaning that the model had to be artificially constrained to produce a curve resembling the observed data. If mortality in a population of ~2000 did not reach a very high proportion the curves obtained in the model, even with small values for *p*, did not resemble those observed in the field (Figure 7H). Similarly it was not possible to model epizootics observed in the large populations that can be present on farms at the earliest commercial life history stage (~100 000 to ~1 000 000) (typically ~2 mm shell length) (data not shown). In all models changes to the incubation period affected only the time scale on the X axis (data not shown).

## Discussion

Mortality data from experimental oysters placed in endemically infected estuaries were analysed to identify important aspects of the epidemiology of OsHV-1 infection. This discussion is structured firstly to address the findings from qualitative analysis and modelling and secondly to address: (i) the source of the infection for index cases and for endemicity, and (ii) the type of transmission of the virus within and between cultivation units.

### Qualitative criteria

Synchronous onset of mortality across cultivation units in a group was observed in only three of the 37 groups, and is best explained by simultaneous point source exposure of the affected units. However, in 92% of the groups there was an asynchronous onset of mortality between cultivation units. This could be explained by separate point source exposures repeated over time, direct transmission from oysters in one cultivation unit to those in another, or both.

The final mortality in all of the units of more than half of the groups was < 70% and in some units < 20% of oysters died suggesting poor transmission between oysters in the same unit even though they were in very close contact. In 41% of the groups there were units in which none of the oysters died, which suggests poor transmission between oysters in different units. In about 40% of the groups there was no evidence of a spatiotemporal pattern consistent with direct transmission between any of the units, but in the other groups direct transmission within a hypothetical incubation period could be inferred. If oyster-to-oyster transmission was efficient over short distances, as soon as the first oysters succumbed to disease in the first unit the virus would spread to oysters in adjacent units because the separation distances were quite short. For trays, clusters of affected units would be observed, and transmission would continue until most oysters in the tray had died. This is shown in Figure 3. Similarly, orderly patterns would be seen along lines of baskets, which were about 10 cm apart, meaning that oysters in adjacent baskets could be no more than about 0.1–1.4 m apart. But the observed patterns of onset of disease were often not orderly. Finally, there was little evidence for expansion of an outbreak in the form of an increasing number of newly affected units, as would occur due to direct transmission between cultivation units. Even when there was still an ample number of unaffected, susceptible units, in 8 of 14 examples the number of new units affected in successive events was lower. There were intervals much longer than the hypothetical incubation period between onsets of mortality in adjacent units, which ruled out direct transmission.

There are a number of potential limitations associated with these observations. Firstly, there was no intensive cultivation of *C. gigas* when we undertook Experiments 1–4; in very densely stocked farming areas there may be a higher pressure of infection, due to the greater biomass, and this might force direct transmission due to higher doses of OsHV-1. Secondly, we assumed the groups to be independent of one another, but there may have been some influence from surrounding groups. Thirdly, as we removed dead oysters it could be argued that we removed the source of virus. However, substantial quantities of OsHV-1 are released into seawater prior to death [43], the viral loads in more than 80% of the dead individuals exceeded 10^4^ copies/mg tissue, and some of the removed oysters were empty shells so virus had already been dispersed into seawater. Therefore it seems improbable that relatively infrequent removal of dead oysters would have prevented direct transmission within units. Fourthly, in Experiments 1 and 2 there were four different age–size combinations within each group, age and size being factors recognised to influence mortality [38], however as the same between unit mortality pattern was observed in Experiment 3 in which only one type of oyster was present in each group, factors other than the age and the size of the oysters were responsible for the observed patterns of transmission. Lastly, we assumed that all oysters were susceptible to OsHV-1; in Experiment 3 which was prolonged, susceptible oysters remained after the first mortality event and succumbed in the next. However, there is genetic variation in susceptibility to OsHV-1 within populations of *C. gigas* [44] which may explain why some individuals survive repeated exposure to OsHV-1 [45].

Notwithstanding the limitations, the qualitative analysis suggested that direct transmission between oysters within a cultivation unit, and between units, if it had occurred, was not very efficient. The limited evidence for direct transmission was not unequivocal because the observed patterns of asynchronous onset could also be explained by successive point source exposures spread over time.

### Modelling

A simple Reed–Frost model was used to examine observed data from Experiments 1, 2 and 3. OsHV-1 infection has a short incubation period, of the order of a few days (Table 2), and oysters that succumb shed the virus prior to death [43]. After death their soft tissues are rapidly removed by autolysis and scavengers in the environment, so the period of transmission would be approximately equal to one incubation period and the assumptions of the model are likely to be valid [41] (see methods section for other assumptions). The Reed–Frost model incorporates all of the myriad host, pathogen and environmental variables (known and unknown) that can influence transmission into a single parameter, the effective rate of contact *p*. This is useful because there are insufficient data for OsHV-1 to parameterise complex transmission variables that might be needed in other types of models. Typical values for *p* used in Reed–Frost models of human childhood viral diseases in populations of 50–100 were 0.01–0.04 [41]. Larger values of *p* (0.16–0.25) were used in models of hepatitis C and influenza transmission [46, 47]. The Reed–Frost models produced for populations of 50 oysters (~50 to ~90 mm length) in segments of trays, using realistic values for input parameters including *p* (0.015–0.035), closely resembled the observed data. Thus in the confined space of the tray segment (40 × 40 × 4 cm) these oysters may have had sufficient exposure to OsHV-1 for direct transmission. However, epidemic curves could not be modelled where mortality commenced and then stalled (Figures 4, 5). Such delays in progression can be better explained by point source exposure events staggered over time, i.e. indirect transmission from successive environmental exposures. The extremely small values for *p* (0.003–0.006) that were needed to produce curves that approximated the observed data for larger populations of oysters in baskets or upwellers meant that the modelling was contrived to obtain a fit and suggested that a process other than direct transmission existed in the real world.

Sartwell’s model was used to assess the incubation period data from the larger populations of small spat in Experiment 4; it required daily mortality observations given the short incubation period of OsHV-1 infection. Based on epidemic theory, incubation periods are not fixed, but rather are normally distributed. Being able to fit a linear relationship between log-time since the start of an epidemic and the resulting cumulative mortality provided strong evidence for a common infection point [39, 40], that is, an environmental source rather than direct transmission between the spat.

Overall the modelling data provided evidence for direct transmission in some circumstances as well as evidence for indirect transmission.

### Source of OsHV-1 for epizootics

Sources of virus for index cases and sources for endemicity and recurrent outbreaks may not be the same.

#### Index cases

Disease due to OsHV-1 microvariants first appeared in *C. gigas* in the northern and southern hemispheres between 2008 and 2010 but it is unclear whether specific strains of OsHV-1 are extending in range globally or whether new pathogenic strains are evolving locally; molecular epidemiological studies may resolve this [48, 49]. There are obvious mechanisms for long distance translocations of marine pathogens that could explain coincident disease emergence, such as shipping and natural rafting of invertebrates in oceanic currents [18–21]. It may be no coincidence that the three regional index cases of disease caused by OsHV-1 in Australia all commenced in estuaries with major commercial shipping operations (Sydney, Hobart, Adelaide). The virus could spread in seawater through movement of particles such as infected larvae [50] with the tide and current from estuaries to oceanic currents and then due to coastal connectivity, move between estuaries [51]. While there is no obvious physical exchange of oysters or used farming equipment between enterprises in Europe and New Zealand/Australia, or between New Zealand and Australia, there is evidence of regional spread of OsHV-1 within Europe and within New Zealand due to oyster translocations by farmers [10, 13]. For many years fresh/frozen *C. gigas* have been imported from New Zealand to Australia for human consumption; uncooked imported seafood has been identified as a significant risk for international pathogen transfer, and may explain the emergence of white spot syndrome virus on shrimp farms in Australia in 2016 [22].

#### Endemicity and recurrent outbreaks

All of the experimental oysters used in this study were free of OsHV-1 infection at the start of each trial, and fomites can be ruled out as a source of virus because clean equipment was used. There was no longer any commercial farming of *C. gigas* that could have acted as a source in either estuary. Therefore the first experimental oysters to become affected by OsHV-1 must have acquired the infection from the environment.

After epizootics in experimental *C. gigas* that were placed in the Georges River estuary Australia there were survivors that had been exposed to OsHV-1 [45] and subclinically infected adult *C. gigas* have also been identified in France [52]. Although latency is a well known mechanism within herpesvirales [2] it was not the reason for the epizootic in the Hawkesbury River estuary, Australia as prevalence did not increase between first detection of OsHV-1 in October 2012 and onset of mortality in January 2013 [16]. Surviving *C. gigas* in the Georges river estuary appeared to be resistant to subsequent exposure events and the prevalence of detectable OsHV-1 in their tissues declined over time so they may not be productive reservoir hosts [45]. In both Australia and Europe there are a range of invertebrate species that harbor OsHV-1 [53, 54], but their role as reservoirs capable of transmitting the virus to *C. gigas* requires confirmation.

Although vertical or pseudo-vertical transmission of OsHV-1 may be possible it has yet to be proven [50], and there are no data for the microvariant strains of OsHV-1 that have been responsible for the epizootics since 2008. Nevertheless, larvae from natural spawning events of wild *C. gigas* could become infected from their parents or from the environment, may be a suitable host for viral amplification and may be capable of transporting the virus during their free-swimming, pelagic stage, which lasts several weeks or if they attach to a floating vehicle.

It seems likely that there is a biological reservoir for OsHV-1 in endemically infected estuaries. There was no farming of *C. gigas* in either estuary when Experiments 1–4 were conducted, therefore the hypothesis of an environmental reservoir is logical but in very densely stocked farming areas an environmental reservoir may be unnecessary to maintain endemicity. It is necessary for a putative reservoir to maintain the virus throughout the winter as outbreaks in farmed oysters are seen only in the warmer months of the year.

### Transmission and disease expression

Following establishment of OsHV-1 in an estuary there are two possible mechanisms of transmission: indirect, which could occur over short or long distances, and direct by close contact. In both human and veterinary medicine contact transmission is understood to mean actual physical interaction or alternatively contact with tissues and secretions from an infected host which can pass a few metres between individuals [32, 33].

Experimentally OsHV-1 can be transmitted through direct contact: i) by inoculation of semi-purified OsHV-1 directly onto or into *C. gigas* [4, 43, 55, 56] ii) by cohabitation of infected and naive oysters in tanks of seawater [8, 26–28]. Apart from Petton et al. [29] who used flow through systems, these experiments were conducted in small aquaria (15–25 L) with short contact distances, minor dilution effects, and long contact times (days). This would favour direct transmission but is not representative of estuaries where dilution rates are extreme, currents and tides move large volumes of water quickly, and scavengers rapidly eliminate tissues from sick and dead oysters thereby removing the source of virus.

In Experiments 1 and 2 there were two to three mortality events spaced over one to 2 weeks and in Experiment 3 there were two main mortality events and a series of minor ones spaced over 5 months. The dead oysters had high viral loads [6, 36–38] and would have released OsHV-1 into surrounding seawater [8, 28, 43, 57] but this did not trigger a propagating epizootic. Surviving oysters often died in subsequent events which indicated they were susceptible and probably had missed out on prior exposure.

Most trays and long lines (34–37 groups) had asynchronous and clustered patterns of onset of mortality among units that can be readily explained by successive point source exposures from the environment. It is likely that variation in the time and dose of exposure of each unit led to different times of onset of mortality for each unit. The very rapid occurrence of high mortality seen in some units was too fast for direct transmission. Similarly, during the 2013 outbreak in the Hawkesbury River, 10 million oysters died in about 3 days, a kill rate too rapid to be explained by direct oyster to oyster transmission [16].

An important piece of evidence consistent with indirect transmission was the detection of OsHV-1 DNA in the incoming seawater before it was detected in the tissues of the spat in Experiment 4 [58]. Similarly, oysters exposed to natural seawater in a holding facility in France succumbed to OsHV-1 [59].

These experiments entailed many variables that may influence disease transmission and expression through the environment–host–pathogen interaction. Could these variables explain the pattern of mortality and affect judgement of the type of transmission? There were different types of cultivation structures, but all made close contact between individual oysters inevitable. Each of the geographic sites had different features but disease due to OsHV-1 had occurred at each site. Perhaps environmental conditions were not always suitable for disease expression after exposure, because in several studies OsHV-1 has been detected in oysters in the absence of mortality [6, 16, 36, 60–62]. Water temperature is important [26, 62–64], but it exceeded the threshold of 16 °C required for OsHV-1 disease expression at all sites and in all experiments. Salinity might play a role [27], but did not vary greatly. Oysters ranged from highly susceptible small spat to larger adults with high filtration rates and potential to filter/ingest pathogens. Perhaps variations in food availability affected transmission because OsHV-1 infection may be acquired during feeding [8]. Numerous environmental parameters influence the filtration rate of oysters in general [65]. Oysters near the edge of a tray segment or basket may have more opportunity to feed than those in the centre of a unit, due to the competition for food [66]. However, the oysters grew and they were freely moveable within the cultivation units. They were mixed by tide, current and physical handling and this would counter spatial effects on feeding, as well as related density effects [8, 29]. The oysters in Experiment 4 were much smaller (5–10 mm) compared to the oysters in the other experiments (40–90 mm); large oysters may release many more virus particles into seawater than do small oysters, have greater filtering capacity [65] and in a confined space this may increase the chance of acquiring an infectious dose and so enable direct transmission. However, it would also facilitate acquisition of OsHV-1 through indirect transmission from an environmental source. It seems unlikely that these factors could have applied consistently at all of the sites, within all of the groups and across time in these experiments. This leaves variation in exposure to OsHV-1 as the most likely reason for the variable patterns of mortality. There is a classical dose–response effect in OsHV-1 infection [43] and some individuals may have survived because they acquired too low a low dose.

Consideration of the role of hydrodynamics is important in exploring the concept of exposure and dose for both direct and indirect transmission. The movements of seawater due to tides and currents may carry viral particles away from individual oysters, reducing the dose they receive, thereby delaying or preventing their mortality. At intermediate scale currents may move viral particles towards or away from an adjacent tray or long line. At larger scale, movements of seawater could carry virus originating from farm or environmental sources over long distances, providing time and opportunity for mixing and interaction of virus with putative carrier particles in the plankton, and leading to disease in other parts of an estuary. Thus local hydrodynamics may favour or prevent exposure, simply by moving “virus clouds” in different directions. Spatial variations in the scale of the “cloud of exposure” influence whether one or more oysters, tray segments, entire trays, baskets or entire long-lines become affected at any given time. This can explain the patchy distribution of disease in the Georges River estuary, Australia at centimeter, metre and kilometer scales [5, 37] and the temporal pattern of disease in the farming and non-farming areas in the Thau Lagoon, France [25]. At each scale, environmental sources of virus may be more important than farm sources, but it might be impossible to distinguish the two when there are obvious farmed populations of *C. gigas* and poorly characterized environmental sources.

That direct transmission could have occurred at a local scale (< 40 cm separation distance), and indirect transmission also at larger scales (> 40 cms between cultivation units, metres between groups, kilometres between sites) is one of the interesting findings of this study. It is consistent with theoretical studies describing pathogen transmission, including in aquatic systems, which hold that transmission can vary with scale [67, 68].

OsHV-1 provides a useful model with which to compare other significant diseases caused by marine herpesviruses. Pilchard herpesvirus (PHV) caused epizootics in sardines *Sardinops sagax neopilchardis* in Australian coastal waters in 2005 and 2008. Sardines are highly mobile filter feeders, dependent on plankton, but appear not to have become infected by a feeding mechanism or particles. Instead, PHV was proposed to have been transmitted directly between fish within schools and between them as fish interchanged between schools, based on computer modelling of disease spread [69]. The mode of transmission of another economically significant marine herpesvirus, *Haliotid herpesvirus 1* of abalone, also appears to be direct. Abalone are grazing animals not filter feeders, and direct contact with the virus shed by other animals is sufficient to establish infection, at least experimentally [70]. *C. gigas* have a sedentary life after larval settlement, and perhaps OsHV-1 has co-evolved efficient mechanisms for indirect transmission to account for this, being assisted by a vector particle and the filter feeding habit of its host.

**Figure 8:**
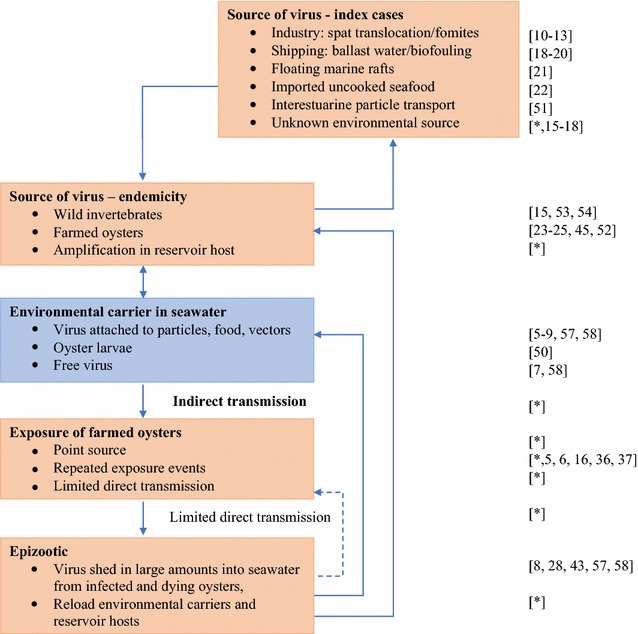
**Proposed conceptual model of OsHV-1 index case source and transmission, leading to endemicity with recurrent seasonal outbreaks.** Following introduction to a naïve population of oysters the virus establishes in an environmental reservoir host, utilises an environmental carrier particle and infects farmed oysters through single or repeated point source exposure. Virus is amplified and shed into seawater, enabling reloading of reservoirs and carrier particles in seawater, leading to repeated point source exposure events. The virus population may expand in reservoirs/vectors. There is limited direct transmission (dashed line), mainly between larger oysters that are in close proximity within cultivation structures. References pertaining to OsHV-1 or to a general process are shown at the right of the figure; * indicates hypothesis or analysis from this paper.

In conclusion, direct transmission of OsHV-1 is inefficient and large scale propagating epizootics were not observed. Instead outbreaks were derived from point source exposures from environmental sources external to the oyster farm, and direct transmission if it occurred was confined to larger oysters within small cultivation units. Cultivation units at farm and bay level were subjected to repeated point source exposures. OsHV-1 may persist subclinically in oysters that have survived an outbreak, and this may allow the virus to overwinter. These oysters could act as a source of infection the following summer and this would require breakdown of the carrier state, viral replication and release of virions, which is associated with pre-clinical and clinical disease. However, the evidence from observational studies is not entirely consistent with this hypothesis and other environmental sources are likely to be important. Therefore research to identify and mitigate environmental sources is a priority. Husbandry approaches to reduce exposure of all oysters and increase the resistance of spat will be more important than reducing contact rates between susceptible oysters through mechanisms such as use of low stocking rates prior to outbreaks, or culling or harvesting in the face of an outbreak. However, devising cultivation structures with physical barriers may be worthwhile to reduce direct transmission between larger oysters, and to influence local hydrodynamics associated with indirect transmission. Biosecurity practices that focus solely on pre-movement testing of oyster spat from hatcheries and prevention of transfers of farm equipment (fomites) are important but are unlikely to fully mitigate the risk of epizootics in unaffected regions. We propose a comprehensive conceptual model of OsHV-1 source and transmission based on these conclusions (Figure 8).

## Abbreviations

OsHV-1: *Ostreid herpesvirus 1*
PCR: polymerase chain reaction
qPCR: quantitative real time PCR
NSW: New South Wales
AVNV: acute viral necrosis virus
SD: standard deviation
DF: dispersion factor
CL: confidence limits
IP: incubation period
PHV: pilchard herpesvirus
